# CCL20/CCR6 expression profile in pancreatic cancer

**DOI:** 10.1186/1479-5876-8-45

**Published:** 2010-05-10

**Authors:** Claudia Rubie, Vilma Oliveira Frick, Pirus Ghadjar, Mathias Wagner, Henner Grimm, Benjamin Vicinus, Christoph Justinger, Stefan Graeber, Martin K Schilling

**Affiliations:** 1 Dept. of General -, Visceral-, Vascular - and Paediatric Surgery, University of the Saarland, 66421 Homburg/Saar, Germany; 2 Institute of Pathology, University of the Saarland, 66421 Homburg/Saar, Germany; 3 Department of Radiation Oncology, Inselspital, Bern University Hospital, and University of Bern, 3010 Bern, Switzerland; 4 Institute of Medical Biometrics, Epidemiology, and Medical Informatics (IMBEI) University of the Saarland, 66421 Homburg/Saar, Germany

## Abstract

**Background:**

CCL20 and its receptor CCR6 have been shown to play a role in the onset, development and metastatic spread of various gastrointestinal malignancies. In this study, the expression profile and clinical significance of the CCL20/CCR6 system in distinct benign, pre-malignant and malignant pancreatic tissues was investigated.

**Methods:**

Using RealTime-PCR, enzyme-linked immunosorbent assay (ELISA), Western Blot and immunohistochemistry, we have analyzed the expression profile of CCL20/CCR6 in resection specimens from patients with chronic pancreatitis (CP) (n = 22), pancreatic cystadenoma (PA) (n = 11) and pancreatic carcinoma (PCA) (n = 25) as well as in the respective matched normal pancreatic tissues.

**Results:**

CCL20 mRNA and protein was weakly expressed in normal pancreatic tissues and CP and PA specimens but significantly up-regulated in PCA (8-fold) as compared to the matched normal tissue (P < 0.05). Moreover, CCL20 mRNA and protein expression was significantly associated with advanced T-category in patients with PCA (P < 0.05). CCR6 mRNA showed a significant up-regulation in all three disease entities as compared to normal tissues (P < 0.05, respectively).

**Conclusion:**

CCL20 and CCR6 were significantly up-regulated in PCA as compared to the normal pancreatic tissue and CCL20 was significantly associated with advanced T-category in PCA patients. This suggests that CCL20 and CCR6 play a role in the development and progression of PCA and may constitute potential targets for novel treatment strategies.

## Background

Pancreatic adenocarcinoma (PCA) is characterized by its late presentation, early and aggressive local and distant metastasis, unresponsiveness to most treatment options and an extremely dismal prognosis [1,2]. Despite of curative surgery the life expectancy of PCA patients is very poor and the 5-year overall survival is less than 20% [3]. Histologically, PCA is characterized by an intense inflammatory reaction and cancer cells shaped like duct-like glandular elements surrounded by fibrosis. As the mortality rate of PCA is virtually equal to its incidence, new strategies and therapies are urgently required to improve the clinical outcome of this disease.

The potential mechanisms and pathways accounting for the aggressive biology of PCA have been intensely investigated pointing to the expression of various pro-angiogenic factors, molecular changes in oncogenes and tumor suppressor genes as well as abnormalities in growth factors and cytokines [4-7]. Currently, accumulating data suggest that chemokines and their receptors play a role in the tumor biology of PCA [8-11] and various other types of cancer [12,13]. A limited number of studies have outlined a role of CCL20 (also termed Macrophage Inflammatory Protein-3α, Larc, or Exodus) in PCA development and progression [14-16]. CCL20 belongs to the family of CC-chemokines but shares only less than 30% identity with other members of this chemokine family. Expression of CCL20 has been reported in macrophages, eosinophils and dendritic cells and it is well established that CCL20 contributes to inflammatory cell recruitment [17]. Only the G-protein coupled 7-transmembrane receptor CCR6, which is also expressed in human dendritic cells, shows a strong interaction with CCL20 [18]. Thus, CCL20 selectively signals through CCR6. Expression of CCL20 has been confirmed in various human cancer entities, such as leukaemia, lymphoma, melanoma, hepatocellular carcinoma, prostate cancer, colorectal adenocarcinoma and lung and oral squamous cell carcinoma [19-22]. Moreover, expression of the CCL20/CCR6 system has been reported in PCA tissues and pancreatic cancer cell lines. Stimulation of the CCR6 bearing PCA cells with CCL20 led to an increased proliferation, migration and invasion and it was postulated that CCL20 may act via autocrine and paracrine mechanisms to contribute to the pathobiology of human PCA [14-16]. Recent studies demonstrated that CCL20 may promote pancreatic tumor cell migration and invasion through the up-regulation of matrix metalloproteinase production [16].

Here, we comparatively investigated the expression profile and clinical significance of the CCL20/CCR6 system in PCA as well as in chronic pancreatitis (CP) and pancreatic cystadenoma (PA) which represent pre-malignant conditions often preceding the development of PCA. Essentially, we report significant CCL20/CCR6 up-regulation in PCA tissues compared to matched normal pancreatic tissues. In addition, we detected a significant correlation of CCL20 expression with advanced T-category in PCA patients suggesting an involvement of CCL20/CCR6 in the development and progression of PCA.

## Methods

### Materials

Surgical specimens and corresponding normal tissue from the same samples were collected from patients who underwent surgical resection at our department between 2002 and 2008.

Informed written consent for tissue procurement was obtained from all patients and the study was approved by the local ethics commission of the Ärztekammer des Saarlandes.

Fifty-eight patients were enclosed in the study, consisting of patients with primary ductal PCA (n = 25), CP (n = 22) and PA (n = 11). In every patient sample the corresponding non-affected normal pancreatic tissue was also analyzed, thus the total sample size was 116. Of the 25 patients with cancer one cancer was classified as pT1, six as pT2, fifteen as pT3 and three as pT4, with positive nodal involvement in 17 cases, according to the UICC TNM classification [23]. No patient had received any kind of neoadjuvant therapy prior to resection. The clinical data and patient characteristics for the different pre-malignant and malignant entities were obtained from a prospective database and are summarized in table 1 and table 2.

**Table 1:** Clinical characteristics of patients with pre-malignant pancreatic diseases

Characteristic	Pancreatic Cystadenoma (n = 11)	Chronic Pancreatitis (n = 22)
Gender		
Male	5	14
Female	6	8
Age (years)		
Median	57.1	53.5
Range	32-73	39-71
Diabetes mellitus		
Positive	2	5
Negative	9	17
Nicotine abuse		
Positive	3	13
Negative	8	9
Alcohol abuse		
Positive	1	8
Negative	10	14

**Table 2:** Clinical characteristics of patients with pancreatic cancer

Characteristic	Pancreatic Cancer (n = 25)
Gender	
Male	17
Female	8
Age (years)	
Median	64.7
Range	42-79
Diabetes mellitus	
Positive	16
Negative	9
Nicotine abuse	
Positive	5
Negative	20
Alcohol abuse	
Positive	3
Negative	22
Largest tumor diamter (cm)	
Median	3.5
Range	1.5-4.7
Tumor (T)-category	
pT1	1
pT2	6
pT3	15
pT4	3
Lymph node metastasis	
Positive	17
Negative	8
Grade	1
G1	6
G2	18
G3	
Vascular permeation	
Positive	6
Negative	19

### Tissue preparation

Tissue specimens were collected immediately after surgical resection, snap frozen in liquid nitrogen and then stored at -80°C until they were processed under nucleic acid sterile conditions for protein extraction. For corresponding normal tissue we used adjacent non-affected tissue from the same resected specimens. All tissues obtained were reviewed by an experienced pathologist and examined for the presence of tumor cells. As minimum criteria for usefulness for our study, we only used tumor tissues in which tumor cells constituted at least > 70% of the tumor biopsy.

### Single-strand cDNA synthesis

Total RNA was isolated using RNeasy columns from Qiagen (Hilden, Germany) according to the manufacturer's instructions. RNA integrity was confirmed spectrophotometrically and by electrophoresis on 1% agarose gels. For cDNA synthesis 5 μg of each patient total RNA sample were reverse-transcribed in a final reaction volume of 50 μL containing 1× TaqMan RT buffer, 2.5 μM/L random hexamers, 500 μM/L each dNTP, 5.5 mM/L MgCl_2_, 0.4 U/μl RNase inhibitor, and 1.25 U/μL Multiscribe RT. All RT-PCR reagents were purchased from Applied Biosystems (Foster City, CA). The reaction conditions were 10 min at 25°C, 30 min at 48°C, and 5 min at 95°C.

### Real-time PCR

All Q-RT PCR assays containing the primer and probe mix were purchased from Applied Biosystems, (Applied Biosystems, Foster City, CA) and utilized according to the manufacturer's instructions. PCR reactions were carried out using 10 μL 2× Taqman PCR Universal Master Mix No AmpErase^® ^UNG and 1 μL gene assay (Applied Biosystems, Foster City, CA), 8 μL Rnase-free water and 1 μL cDNA template (50 mg/L). The theoretical basis of the qRT assays is described in detail elsewhere [24]. All reactions were run in duplicates along with no template controls and an additional reaction in which reverse transcriptase was omitted to assure absence of genomic DNA contamination in each RNA sample. For the signal detection, ABI Prism 7900 sequence detector was programmed to an initial step of 10 min at 95°C, followed by 40 thermal cycles of 15 s at 95°C and 10 min at 60°C and the log-linear phase of amplification was monitored to obtain C_T _values for each RNA sample.

Gene expression of all target genes was analyzed in relation to the levels of the slope matched housekeeping genes phosphomannomutase (PMM1) and cyclophilin C (CycC) [25]. Analysis was performed using the delta CT method and samples were normalized to the control tissue sample. Hence, the normal tissue became the 1 × sample, and all other quantities were expressed as an n-fold difference relative to this tissue.

### Isolation of total protein

Protein lysates from frozen tissue were extracted with the radioimmunoprecipitation (RIPA) buffer containing Complete, a protease inhibitor cocktail (Roche, Penzberg, Germany). Total protein quantification was performed using the Pierce BCA protein assay reagent kit (Pierce, Rockford, Ill., USA).

### Sandwich-Type Enzyme-Linked Immunosorbent Assay

The chemokine protein levels in the different tissue lysates were determined by sandwich-type enzyme-linked immunosorbent assays (ELISA) according to the manufacturer's instructions. Samples were assayed in duplicate with all values calculated as the mean of the two measurements. CCL20 levels were assayed using a validated commercial ELISA (Duo Set R&D Systems, DY360, Minneapolis, Minn., USA). The absorbance was read at 450 nm in a 96-well microtiter plate reader. The chemokine concentration from each tissue lysate was normalized to the total protein content of each sample.

### Immunohistochemistry

Operative specimens were routinely fixed in formalin and subsequently embedded in paraffin. Before staining, 4-μm thick paraffin-embedded tissue section were mounted on Superfrost Plus slides, deparaffinized and rehydrated in graded ethanol to deionized water. The sections were microwaved with an antigen retrieval solution (Target Retrieval, Dakocytomation, Carpinteria, CA, USA) and after blocking of endogenous peroxidase activity with 3% hydrogen peroxide, the sections were further blocked for 30 min at room temperature with normal rabbit serum. Overnight incubation at 4°C with primary goat polyclonal anti-human CCL20 antibody (15 μg/ml, AF254, R&D Systems, Minneapolis, Minn., USA) was followed by incubation of secondary biotinylated rabbit anti-goat IgG antibody and the avidin-biotin-peroxidase reaction (Vectastain ABC ELITE Kit, Vector Laboratories, Burlingame, CA, USA). After colour reaction with aminoethylcarbazide solution (Merck, Darmstadt, Germany), tissues were counterstained with haematoxylin. Negative controls were performed in all cases omitting primary antibody.

### Western Blot Analysis

Total protein (25 μg/lane) was separated by SDS-PAGE using a 10% gel and blotted onto nitrocellulose membranes (Hybond ECL, Amersham Biosciences, Piscataway, NJ, USA). Membranes were blocked by incubation in Tris-buffered saline (TBS) containing 5% nonfat dry milk and 0.1% Tween 20 for 2 h at room temperature and then incubated overnight at 4°C with goat anti-human CCR6 antibody (diluted 1:500, C2099-70B, Biomol, Hamburg, Germany). Blots were then washed and incubated at room temperature for 1 h with donkey anti-goat HRP antibody (diluted 1:5000, sc-2056, Santa Cruz Biotechnology, Santa Cruz, CA USA). Bands were visualized by ECL Western blotting analysis systems (Amersham Biosciences, Piscataway, NJ, USA). The human cell lysate HL-60 (sc-2209, Santa Cruz Biotechnology, Santa Cruz, CA, USA) served as positive control. Quantification of figure four has been performed on three independent samples using image J software.

### Calculations and Statistical Analysis

All chemokine concentrations are presented as mean and SEM (standard of the mean). All statistical calculations were done with the MedCalc (MedCalc software, Mariakerke, Belgium) software package [26]. The parametric Student's t-test was applied, if normal distribution was given, otherwise, the Wilcoxon's rank sum test was used. T-category was dichotomized (pT1 - pT2 vs. pT3 - pT4). P-values < 0.05 at a two-sided level of α < 0.05 were considered significant.

## Results

### CCL20/CCR6 mRNA expression in benign, pre-malignant and malignant pancreatic tissues

**Figure 1:**
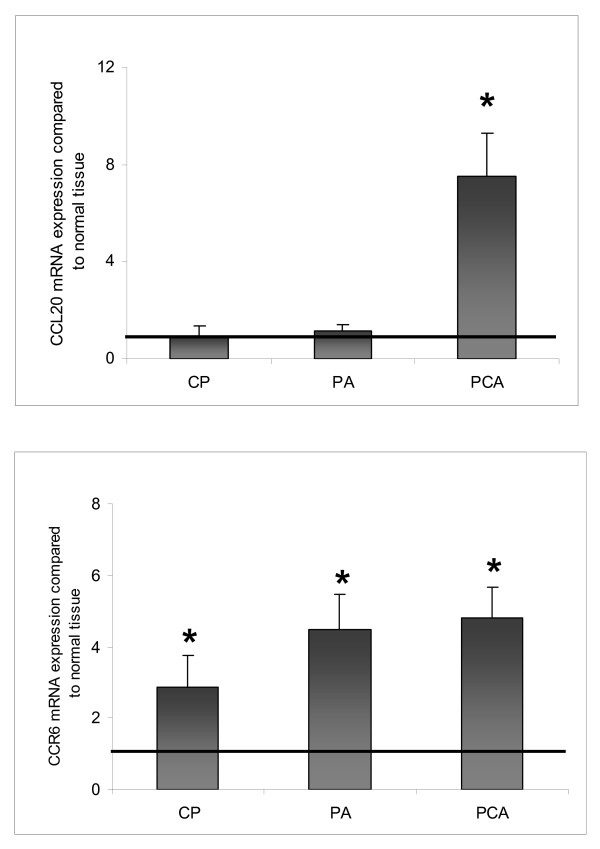
**CCL20/CCR6 mRNA expression in pancreatic diseases**. Gene expression of [A] CCL20 and [B] CCR6 in chronic pancreatitis (CP, n = 22), pancreatic cystadenomas (PA, n = 11), pancreatic carcinoma (PCA, n = 25) compared to matched normal pancreatic tissues as determined by Q-RT-PCR. Q-RT-PCR data are expressed as mean +/- SEM, *P < 0.05. Fold increase above 1 indicates CCL20/CCR6 up-regulation compared to normal tissues.

CCL20 mRNA was weakly expressed in the normal pancreatic tissue as well as in PA and CP specimens. In PCA specimens CCL20 mRNA showed a significant 8-fold up-regulation as compared to the matched normal tissues (P < 0.05) (Figure 1A). As shown in Figure 1B CCR6 mRNA expression was significantly up-regulated in all 3 disease entities (P < 0.05, respectively) as compared to the normal tissue, with PCA and PA specimens showing a 4-fold and CP tissues showing a 3-fold up-regulation, respectively.

### CCL20/CCR6 protein expression in benign, pre-malignant and malignant pancreatic tissues

**Figure 2:**
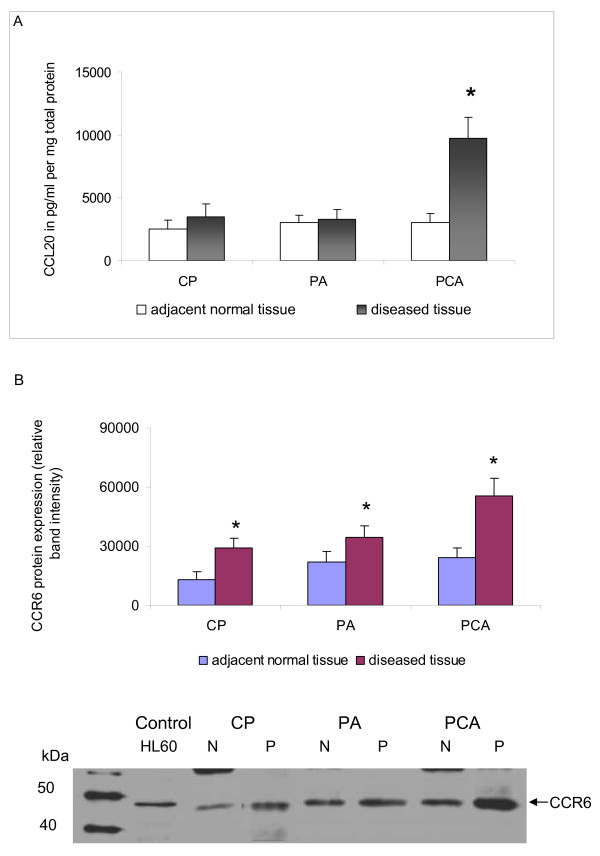
**CCL20/CCR6 protein expression in pancreatic diseases**. [A] CCL20 protein concentrations (pg/ml pro mg total protein) in chronic pancreatitis (CP, n = 22), pancreatic cystadenomas (PA, n = 11), pancreatic carcinoma (PCA, n = 25) compared to the matched normal pancreatic tissue levels (mean ± SEM), * p < 0.05. [B] Expression of chemokine receptor CCR6 in CP, PA and PCA as determined by Western blot analysis. Total cell lysates of tumor tissues of representative patients of each disease entity were immunoblotted with antibodies specifically recognizing chemokine receptor CCR6. Acute leukemia cell line HL60 served as a positive control for the detection of CCR6. Quantification has been performed using image J software * p < 0.05.

CCL20 protein was weakly expressed in normal pancreatic tissue and in PA and CP specimens. In PCA the CCL20 protein expression showed a significant 3-fold up-regulation compared to the matched normal pancreatic tissues (P < 0.05) (Figure 2A).

As assessed by western blot analysis CCR6 protein expression was detectable in all pancreatic disease entities under investigation, namely in CP, PA and PCA tisssue specimens as shown for representative patients in Figure 2B. However, band intensity was significantly higher in the diseased tissues (P < 0.05) and showed the highest value in PCA tissues (Figure 2B).

**Figure 3:**
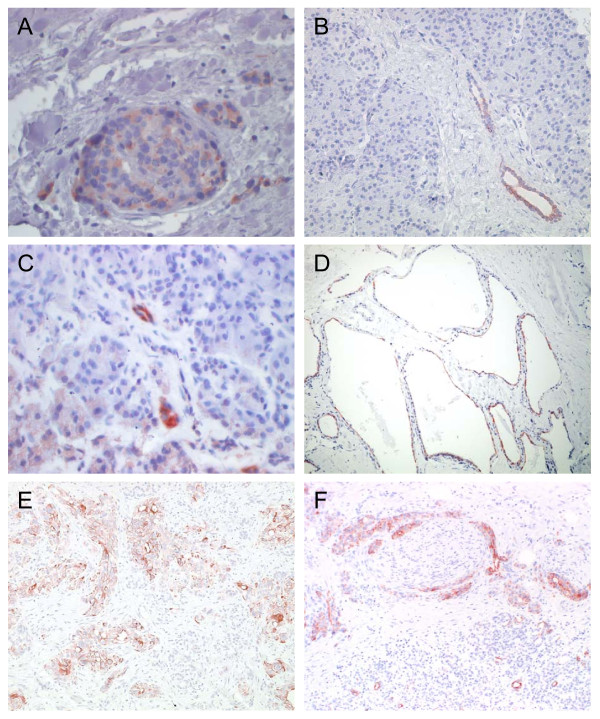
**Results of anti-CCL20 immunohistochemistry in normal and diseased pancreatic tissues**. Representative example of CCL20 expression in [A,B] pancreatic islet cells and epithelial cells of pancreatic ducts, [C] necrotic parenchyma and epithelial cells of pancreatic ducts in chronic pancreatitis tissues [D] epithelial cells of the characteristic net-like structures of pancreatic cystadenoma [E,F] cytoplasms of ductal epithelial cancer cells and in infiltrates of perineural sheaths. Anti-CCL20 goat anti-human, 75 μg/ml (MIP-3α; R&D Systems; Minneapolis, MN, USA. Avidin Biotin Complex (ABC) Method. (original magnification: × 200 and 400, respectively).

Using immunohistochemistry CP, PA and PCA specimens along with the corresponding normal tissues were evaluated for CCL20 expression (Figure 3). CCL20 signals were detected in all CP, PA and PCA specimens under investigation. In normal tissues, CCL20 staining was primarily found in pancreatic islet cells and rather sporadically in epithelial cells of pancreatic ducts as shown in Figure 3A and 3B. In CP tissues immunoreactive CCL20 signals were detected primarily in acinar parenchyma deformed by necrosis and sporadically in some epithelial cells of pancreatic ducts (Figure 3C). Likewise, CCL20 staining was found in epithelial cells of the characteristic net-like structures of PA tissues (Figure 3D). In PCA tissues CCL20 immunoreactivity was detected in the cytoplasms of ductal epithelial cancer cells (Figure 3E). Moreover, CCL20 signals were detected in infiltrates of perineural sheaths as shown in Figure 3F.

### T-Category dependent CCL20 expression in PCA

CCL20/CCR6 mRNA and protein expressions in PCA were compared to several clinicopathological factors such as TNM stages, age, lymphatic and vascular invasion or pre-existing conditions like cirrhosis or fibrosis.

**Figure 4:**
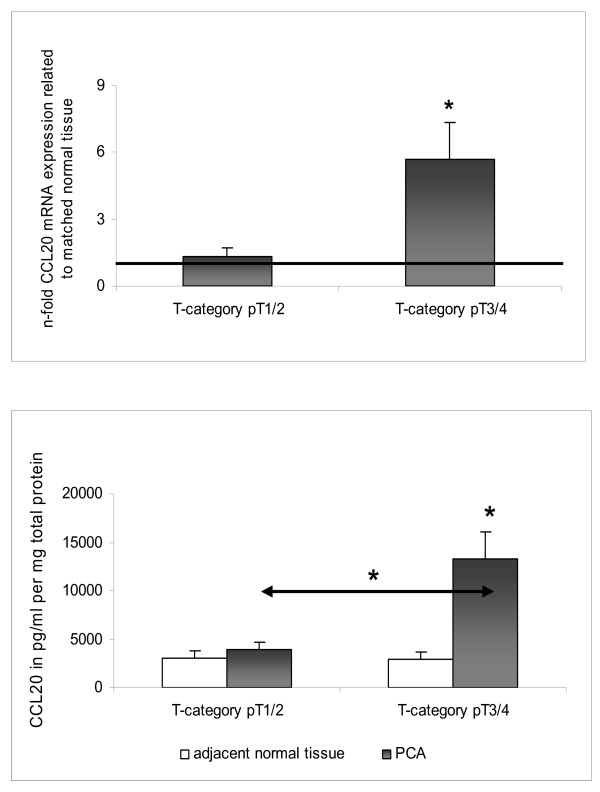
**Expression of CCL20 in different tumor categories of PCA as determined by [A] Q-RT-PCR and [B] ELISA**. mRNA and protein expression profiles of CCL20 (pg/ml pro mg total protein) were measured in PCA tissues and matched normal tissues in pT1 + pT2 (n = 11) (A) and pT3 + pT4 (n = 14) (B), respectively. For Q-RT-PCR data fold increase above 1 indicates CCL20 up-regulation in PCA compared to normal tissues. All data are expressed as mean ± SEM, * p < 0.05

Clinical validation of CCR6 expression showed no significant association with any of the clinicopathological factors tested. However, CCL20 mRNA and protein expressions in PCA patients were significantly associated with advanced T-category (p < 0.05) (Figure 4).

## Discussion

In order to evaluate the potential contribution of CCL20/CCR6 in development and progression of PCA, we analyzed the mRNA and protein expression profiles of the CCL20/CCR6 system in patients with PCA as well as in patients with PA and CP, which represent pre-malignant diseases which often precede the formation of pancreatic malignancies [27,28]. Originally, CCL20 and CCR6 were demonstrated to play a role in inflammatory responses [29,30]. Thus, CCL20 was presented as a potent chemoattractant for immature dendritic cells (DCs) and their precursors to sites of potential antigen-entry [31]. In PCA, co-expression of CCL20 and CCR6 was reported in PCA tissues and in cultured human PCA cell lines. It has been demonstrated by others that stimulation of CCR6 bearing PCA cells with CCL20 led to increased proliferation, migration and invasion [14-16].

In accordance with earlier studies [14] reporting a faint but distinct CCL20 transcript in northern blot analysis of normal human pancreatic tissues, we observed low CCL20 mRNA and protein expression levels in the normal pancreatic tissues analyzed. In PCA tissues the CCL20 transcript was detected in moderate to high levels [14] and expression of the CCL20 protein was observed in cancer cells within the pancreatic tumor mass [16]. In accordance with these studies, we have observed a significant up-regulation of CCL20 mRNA and protein expression in PCA. Interestingly, comparing several clinicopathological factors to CCL20 mRNA and protein expression levels we found a significant correlation with advanced T-category pointing to a role for CCL20 and CCR6 in progression of PCA.

By immunohistochemistry we detected in CP immunoreactive CCL20 signals primarily in necrotic parenchyma and sporadically in some epithelial cells of pancreatic ducts, whereas in PA CCL20 staining was found in epithelial cells of the net-like structures of PA tissues and in pancreatic islet cells. In the PCA tissues we observed CCL20 immunoreactivity in the cytoplasms of ductal epithelial cancer cells, in infiltrates of perineural sheaths and also in tumor-associated macrophages. Others have reported that CCL20 could not be detected by immunohistochemistry in normal pancreatic tissue [16].

In CP and PA specimens, the CCL20 mRNA and protein expression was weak, comparable to matched normal tissues. CP has been suggested as an independent risk factor for the development of pancreatic cancer [28,32]. However, the risk of developing PCA in CP is also related to other factors such as age, the progression of molecular mutations, smoking, obesity and alcohol abuse [33,34]. Since the CCL20 expression profile in CP is entirely inconspicuous, a putative role of CCL20 and CCR6 in the process of progression from CP into PCA can be ruled out. Likewise, we observed an entirely inconspicuous CCL20 expression profile in PA, a pancreatic disease also suspected to precede the development of PCA. Thus, up-regulation of CCL20 mRNA and protein is restricted to PCA tissues whereas CCL20 is not up-regulated by PA and CP. In contrast, CCR6 mRNA was not exclusively up-regulated in PCA but also in PA and CP as compared to the matched normal tissues. Up-regulation of CCR6 expression has been observed in various cancer entities [19,20,22,35]. However, also in inflammatory diseases up-regulation of CCR6 expression has been reported [36-38].

In summary, our findings suggest that CCR6 transcripts are up-regulated in PCA as well as in the pre-malignant pancreatic diseases CP and PA. In contrast, up-regulation of CCL20 mRNA and protein is restricted to PCA. Our results provide evidence that CCL20 expression is correlated with advanced T-category representing advanced PCA. On the basis of our findings and the current literature we conclude that CCL20 and CCR6 are involved in the development and progression of PCA and may constitute potential targets for novel treatment strategies.

## Conclusions

The results of this manuscript show that CCL20 and its corresponding receptor CCR6 are significantly up-regulated in patients with pancreatic cancer (PCA) and that CCL20 is significantly associated with advanced T-category in those patients. This suggests that CCL20 and CCR6 play a role in the development and progression of PCA. Thus, inhibition of CCR6 signalling or neutralization of CCL20 or inhibition of its production and activity may be useful in preventing further progression of the disease and may be a future basic treatment strategy in the management of PCA.

## Competing interests

The authors declare that they have no competing interests.

## Authors' contributions

All authors read and approved the final manuscript

All authors read and approved the final manuscript. CR is responsible for the design of the study, interpretation of the results and drafted the manuscript. VOF took part in all experimental elements, performed the ELISAs and participated in scientific discussions and interpretation of the results. MW examined the tissue sections for the presence of tumor cells, histopathologically confirmed all tissues under investigation, participated in scientific discussions and data interpretation. PG participated in scientific discussions and is responsible for the critical assessment and revision of the manuscript. HG collaborated in all the experimental elements. BV performed the western blots and contributed to scientific discussions and data interpretation. CJ provided clinical information and SG participated in the statistical analysis. MS is responsible for the provision of all the patient material and clinical information, participated in scientific discussions, data interpretation and revision of the manuscript.
